# Natural Nacre-Derived Biomimetic Materials for In Vivo Bone Regeneration

**DOI:** 10.3390/biomimetics11020114

**Published:** 2026-02-04

**Authors:** Pierre-Yves Collart-Dutilleul, Naveen Fatima, Richard Younes, Frédéric Cuisinier, Véronique Barragan-Montero, Alban Desoutter

**Affiliations:** 1 Laboratory Bioengineering Nanosciences (LBN), University of Montpellier, 34193 Montpellier, Francerichard.younes@umontpellier.fr (R.Y.); frederic.cuisinier@umontpellier.fr (F.C.); veronique.montero@umontpellier.fr (V.B.-M.); alban.desoutter@umontpellier.fr (A.D.); 2 Department of Odontology, Montpellier University Hospital, CHU Montpellier, 34193 Montpellier, France

**Keywords:** bone regeneration, nacre proteins, porous silicon microparticles, critical-size defect, osteoinduction, biomimetic materials, tissue engineering

## Abstract

Bone regeneration in critical-size defects requires biomaterials that provide both structural support and appropriate osteoinductive cues. Natural nacre contains an organic matrix rich in acidic macromolecules with reported osteogenic activity; however, its in vivo regenerative potential remains insufficiently explored. This study evaluated the bone regenerative capacity of nacre-derived materials alone and combined with oxidized porous silicon microparticles (pSi-MP), a bioactive material known to release silicic acid and support mineralized tissue formation. Critical-size defects were created in four caudal vertebrae of Wistar rats and filled with nacre, pSi-MP, a nacre–pSi composite, or left empty. After 60 days, bone formation was assessed using micro-computed tomography and non-decalcified histology. Empty defects failed to regenerate, whereas nacre and pSi-MP individually promoted partial mineralized tissue deposition. The nacre–pSi composite produced the most extensive repair, showing near-complete defect bridging, higher bone mineral density, and seamless integration of particles within newly formed bone. No inflammation or adverse reactions were observed, and osteoid deposition occurred directly on material surfaces. These findings demonstrate that nacre-derived materials exert intrinsic osteogenic effects in vivo and that combining nacre with porous silicon yields a synergistic response that significantly enhances bone regeneration. The composite represents a promising candidate for future bone repair strategies.

## 1. Introduction

Natural composites found in biological organisms have long inspired the development of advanced biomaterials for tissue engineering. These composites typically integrate a stiff ceramic phase and a softer organic phase arranged into hierarchical, multi-scale architectures that optimize strength, damage tolerance, and bioactivity. Among them, nacre (mother-of-pearl) stands out as one of the most mechanically efficient and biologically intriguing natural materials. Nacre is composed of ~95% aragonitic calcium carbonate platelets and ~1–5% organic macromolecules forming a layered “brick-and-mortar” architecture with exceptional mechanical performance, including tensile strengths exceeding 120 MPa and an elastic modulus of ~70 GPa—values superior to mammalian cortical bone [1]. This combination of stiffness, toughness, and biological functionality has motivated extensive research into nacre-inspired biomaterials [2,3].

Structurally, nacre is organized into microscopic aragonite tablets stacked in parallel laminae. These tablets are bonded by an organic matrix composed of chitin, silk-like proteins, acidic glycoproteins, and polysaccharides [4]. While the overall architecture is conserved across molluscan species, the arrangement differs between bivalves, gastropods, and cephalopods, reflecting distinct crystal growth modalities [5]. Critically, the organic matrix plays an active role in controlling mineral nucleation, crystal orientation, and mechanical behavior. Water-soluble nacre molecules—known as water-soluble matrix fractions—contain acidic, aspartate-rich proteins with demonstrated bioactivity on mammalian cells. Several studies have shown that nacre extracts stimulate osteoblast adhesion, proliferation, differentiation, and matrix mineralization [6,7,8]. For example, water soluble matrix components upregulate ALP activity, promote collagen type I deposition, modulate ERK-dependent signaling, and stimulate mineralization nodules in vitro [9,10]. Other studies have suggested that nacre’s organic molecules may exert bone morphogenetic-like signaling, including peptides capable of inducing osteoblast maturation and inhibiting osteoclastogenesis. [11,12].

Recent advances in biomimetic materials science have reinforced this concept, demonstrating that hierarchical integration and multiscale organic–inorganic coupling—rather than composition alone—are critical determinants of functional performance in nacre-inspired systems [13,14,15,16,17]. In particular, multiscale crosslinking strategies that emulate the molecular-to-macroscopic integration observed in nacre and bone have been shown to dramatically enhance toughness, structural integrity, and biological responsiveness in synthetic composites.

Beyond structural considerations, nacre’s biological relevance for bone regeneration largely arises from its organic matrix proteins. Water-soluble nacre components, enriched in acidic and aspartate-rich macromolecules, have been shown to exert direct biological effects on mammalian cells. Experimental studies consistently report enhanced osteoblast and mesenchymal stem cell adhesion, proliferation, and osteogenic differentiation, with upregulation of key markers such as alkaline phosphatase, collagen type I, osteocalcin, Runx2, and BMP-related signaling pathways. More recent work has further highlighted the capacity of nacre-derived organic components to modulate cell–matrix interactions, cytoskeletal organization, and mineral nucleation dynamics, reinforcing the view that nacre proteins function as instructive biochemical cues rather than inert additives [10,13,18,19,20]. Nacre powders and nacre-containing scaffolds have been shown to enhance bone repair in calvarial, mandibular, and long bone defects [7,21,22]. In a large-animal minipig model, nacre fragments enhanced bone–implant contact and bone volume density around titanium implants [22]. In sheep, implants coated with nacre proved safe and osteoconductive without inflammatory complications [23]. More recently, researchers have developed nacre-mimetic scaffolds that replicate the hierarchical lamellar architecture using hydroxyapatite, chitosan, cellulose nanofibers, or gelatin [15,16,24,25,26]. These scaffolds promote osteogenesis, angiogenesis, and immunomodulation via pathways such as TGF-β/Smad, ERK, and OPG/RANKL, demonstrating that nacre’s structural principles and protein components provide a powerful template for bone tissue engineering [24,26,27].

A central element explaining nacre’s bioactivity lies in its organic matrix proteins, which act as biochemical regulators of mineral deposition in mollusks and as osteogenic cues in mammalian systems [4]. Water-soluble nacre proteins have been reported to stimulate human mesenchymal stem cell (MSC) differentiation, increase alkaline phosphatase activity, modulate the cytoskeleton, and enhance mineralized matrix formation [9,10,21]. Nacre’s organic extracts have also demonstrated anti-inflammatory properties and pro-angiogenic activity [12,24]. Taken together, contemporary research supports the view that nacre’s regenerative potential arises from the synergistic interplay between its organic matrix and mineral phase, rather than from either component alone. This dual structural–biochemical functionality places nacre-derived materials within a broader class of next-generation biomimetic systems that leverage hierarchical organization, controlled degradation, and biologically active interfaces to guide tissue regeneration. Within this framework, investigating nacre-derived materials in combination with complementary bioactive carriers represents a rational strategy to enhance bone regeneration by integrating instructive biochemical cues with tailored material architectures.

In parallel, porous silicon (pSi) has emerged as a highly promising biomaterial for bone repair owing to its nanoscale architecture, high specific surface area, and finely tunable degradation behavior [28,29]. Under physiological conditions, pSi undergoes progressive hydrolytic resorption into orthosilicic acid, a naturally occurring and non-toxic silicon species, with degradation kinetics that can be precisely adjusted from hours to several months through control of pore size, porosity, doping level, and surface oxidation or functionalization. This controlled biodegradation enables temporal matching between scaffold resorption and new tissue formation [30,31]. At the surface level, oxidized and silanized pSi provides a hydrophilic, nanostructured interface that promotes protein adsorption, cell adhesion, and cytoskeletal organization, while also favoring calcium phosphate nucleation and mineralized matrix deposition. Numerous in vitro studies have demonstrated that pSi supports adhesion, proliferation, and osteogenic differentiation of mesenchymal stem cells without inducing cytotoxic or genotoxic effects. Importantly, these properties translate in vivo: oxidized pSi particles exhibit controlled long-term dissolution, sustained silicic acid release, and excellent biocompatibility, integrating progressively into newly formed bone and enhancing mineralization compared with non-oxidized or bulk silicon controls [29,31,32]. In rat caudal vertebra critical-size defect models, pSi has been shown to act as an osteoconductive and bioactive microcarrier, improving bone healing kinetics while remaining non-inflammatory and fully bioresorbable, thereby positioning pSi as a robust platform for bone tissue engineering and for combination with complementary biomimetic bioactive components. [31,32,33].

Combining nacre proteins with porous silicon is therefore scientifically compelling: nacre proteins provide biochemical osteoinductive cues, and pSi provides structural support, controlled degradation, and silicic acid–driven osteogenesis. Together, they may create a synergistic environment for bone repair, with pSi acting as a bioactive carrier for delivering nacre molecules directly to the bone defect. Despite decades of attention on nacre-inspired materials and nacre extracts, very few studies have evaluated the in vivo regenerative potential of purified, natural nacre proteins themselves. Most existing work uses nacre powders, fragments, or nacre-like composites—not isolated molecular fractions. Moreover, no study has examined the potential synergy between nacre proteins and porous silicon microcarriers, despite the highly complementary mechanisms of action. The nacre-derived material was intentionally used in its native organic/mineral context rather than as a purified or molecularly defined protein fraction. While extensive biochemical and proteomic characterization of nacre organic matrices has been reported in previous studies, the objective of the present work was not to investigate the molecular identity or individual bioactivity of specific nacre proteins. Instead, we aimed to evaluate the in vivo biological response elicited by a biomimetic nacre-derived composite that preserves the natural association between organic components and the mineral phase. This approach was chosen to better reflect the hierarchical and multifunctional nature of nacre as a biological material, and to assess its regenerative potential in a physiologically relevant configuration.

Building on these insights, the present study aimed to investigate the in vivo bone regenerative potential of a nacre-derived biomimetic material, used alone or in combination with oxidized pSi microparticles, in a critical-size vertebral defect model in rats. While the osteogenic activity of nacre-derived components and the bioactivity of porous silicon have been independently reported, their combined use as a composite system integrating biochemical osteogenic cues with a degradable, high-surface-area carrier has not been previously evaluated in vivo. The novelty of this work lies in assessing whether preserving the native organic/mineral context of nacre, rather than using purified proteins, can synergize with the structural and controlled-resorption properties of porous silicon to enhance bone regeneration. By directly comparing nacre-derived material, porous silicon microparticles, and their combination within the same experimental framework, this study provides a proof-of-concept evaluation of a biomimetic composite strategy designed to promote osteogenesis through the convergence of structural and biological signals. The outcomes of this work aim to inform the rational design of multifunctional biomaterials for future bone regenerative applications.

## 2. Materials and Methods

### 2.1. Recovery of Nacre-Derived Materials

Nacre was collected from *Crassostrea gigas* oyster shells obtained in the Thau Lagoon (Hérault, France). Approximately 1 kg of shells was manually cleaned under running water, and residual organic material was removed using a stiff brush. Shells were then subjected to a controlled decalcification–recalcification protocol adapted from established nacre matrix extraction procedures as reported in previously published works [34,35]. Nacreous shells were first mechanically cleaned and immersed in a sulfamic acid solution (pH 0–1) for 12 h to remove external contaminants and residual organic debris. Following acid treatment, the shells were thoroughly rinsed with deionized water, and this cleaning cycle was repeated daily for seven consecutive days. After the final acid treatment, shells were extensively rinsed until neutral pH was reached. The cleaned nacreous material was then air-dried at room temperature and mechanically fragmented using a ball-mill grinder (Retsch MM400, Haan, Germany), yielding approximately 100 g of nacre powder. Laser diffraction analysis revealed a particle size distribution ranging from 1 to 5 µm [36]. Particles were sterilized in 90% ethanol for 15 min, before rinsing in PBS

To preserve the bioactive organic matrix while maintaining the native mineral context, the nacre powder was processed following established decalcification–recalcification protocols adapted from previously published nacre matrix extraction procedures. Briefly, controlled partial decalcification was achieved under mild acidic conditions, allowing solubilization of a fraction of calcium carbonate while retaining water-soluble and acid-soluble organic matrix components. The suspension was subsequently rinsed repeatedly with deionized water to remove residual acid and soluble salts. Recalcification was then achieved by restoring near-neutral pH conditions, enabling controlled reprecipitation of calcium carbonate in the presence of the organic matrix and preserving the intimate association between mineral and organic phases characteristic of native nacre.

The resulting material corresponds to a whole nacre-derived organic/mineral composite rather than a purified protein extract. The protein content of the powder was approximately 10% (*w*/*w*), consistent with reported proportions of the nacre organic matrix. This whole nacre powder—containing the native water-soluble organic fraction associated with calcium carbonate—was used for all in vivo experiments, in accordance with biomimetic strategies described in the literature.

In the study presented here, the term “nacre-based materials” refers to a composite system composed of calcium carbonate associated with its native organic matrix, rather than to purified nacre proteins or a defined protein extract. The mineral phase was intentionally preserved in order to maintain the natural organic–inorganic association characteristic of nacre and to reflect a biomimetic design strategy. As such, the calcium carbonate component is expected to contribute to osteoconductive behavior and radiopacity, particularly in micro-CT analyses, while the associated organic fraction provides biologically active cues that may support osteogenic processes. The present work therefore evaluates the combined biological and structural effects of a nacre-derived organic/mineral material, and the respective contributions of these components are discussed accordingly.

### 2.2. Production of Porous Silicon Microparticles

Porous silicon microparticles were produced following an established electrochemical etching protocol for biomedical-grade pSi preparation. Boron-doped crystalline silicon wafers (p++ type, resistivity 0.0012 Ω·cm, Siltronix, Archamps, France) were etched in a custom-made Teflon electrochemical cell. Porosity was generated under a constant current density of 200 mA·cm^−2^ for 30 min in a 3:1 (*v*/*v*) solution of hydrofluoric acid (HF, 48%) and ethanol.

The resulting porous layer was detached from the bulk wafer using a secondary etch step in 3.1% HF in ethanol at 4 mA·cm^−2^ for 4 min. Detached layers were rinsed in ethanol and fragmented into microparticles by ultrasonication for 5 min at 25 Hz in an ultrasonic bath. To slow biodegradation kinetics in vivo, pSi microparticles were thermally oxidized in ambient air at 400 °C for 1 h, as previously described [31]. Particles were sterilized in 90% ethanol for 15 min, before rinsing in PBS. These parameters led to particles of 70–80% porosity, with pores diameters ranging from 30 to 40 nm, and particles size of 50 to 70 µm [29,32]. Oxidized pSi particles were then stored in sterile conditions until further use.

### 2.3. In Vivo Experiments

#### 2.3.1. Ethical Approval

All animal procedures complied with European Directive 2010/63/EU for animal experimentation and were approved by the institutional ethics committee (approval number #40328-2023). Six adult male Wistar rats (350–400 g) were used.

#### 2.3.2. Rat Caudal Vertebra Critical-Size Defect Model

A previously validated vertebral critical-size defect model was adapted to assess the osteogenic effect of nacre-derived materials alone or combined with porous silicon microparticles. Animals were anesthetized via intraperitoneal injection of ketamine (40 mg·kg^−1^) and xylazine (9 mg·kg^−1^). After disinfection of the tail, a dorsal midline incision was made from caudal vertebrae Cd31 to Cd35. Skin and periosteum were gently retracted under continuous PBS irrigation to expose the dorsal surface of four consecutive vertebrae [33].

**Figure 1:**
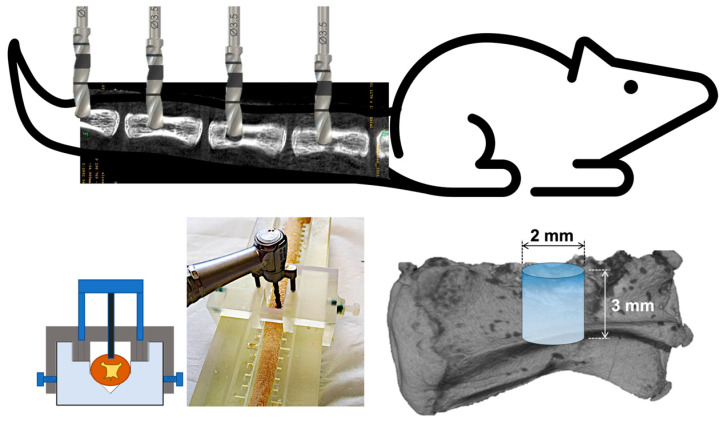
Schematic illustration of the bone defect model. Four defects are drilled in caudal vertebrae, using a stainless custom-made surgical guide. The cylindrical drilled defects have a size of 2 mm of diameter and 3 mm of height.

Using a custom stainless-steel surgical guide for optimal reproducibility, an intraosseous cylindrical defect was drilled in each vertebra using a low-speed dental handpiece under constant irrigation. The created defect was of 2 mm diameter and 3 mm height. A schematic illustration of the surgical procedure is presented in Figure 1. In each rat, the four defects were filled with 20 µL of studied materials to completely seal the drilled bone hole. The following experimental conditions, depending on the study design, were:

- Nacre powder
- pSi microparticles (oxidized or non-oxidized)
- Nacre + pSi composite (1:1 *w*/*w*)
- Empty defect (control)

Wounds were closed in layers using resorbable sutures (Vicryl^®^ 4-0, Ethicon, Raritan, NJ, USA). Animals recovered under a warming lamp. An injection of 0.2 mg meloxicam/kg body weight was done after 4 h and after 24 h. The rats were kept in light controlled, air-conditioned rooms, fed ad libitum, and were monitored daily during the cicatrization period.

No intermediate imaging or biochemical analyses were performed, as the study design focused on end-point evaluation at 60 days to assess final defect bridging and mineralized tissue formation.

#### 2.3.3. Euthanasia and Tissue Collection

Animals were sacrificed at 60 days post-surgery using CO_2_ overdose followed by cervical dislocation. The entire tail was excised, fixed in 4% paraformaldehyde (PFA) for 48 h at room temperature, and stored in PBS at 4 °C prior to micro-CT and histological analysis. Each animal served as its own internal control (four defects per rat).

### 2.4. Micro-CT Analysis

Longitudinal radiographic assessment of bone regeneration was performed at Day 0 (post-surgery) and Day 60 using a SkyScan 1076 system (Bruker microCT, Billerica, MA, USA). Samples were mounted on a 66-mm specimen holder and scanned at a voxel size of 18 µm. A 1.0-mm aluminum filter was used to select the harder portion of the X-ray spectrum. Projections were acquired with an angular step of 0.30°, with frame averaging set to 2. The 360° rotation mode was disabled. Projection images were exported in TIFF format. Regions of interest (ROI) were manually defined around each defect, and quantitative parameters (bone volume fraction, bone mineral density) were extracted using ImageJ (NIH, Bethesda, MD, USA) with the BoneJ plugin.

### 2.5. Histology

Non-decalcified mineralized tissues were processed using the Technovit^®^ 9100 (Kulzer, Hanau, Germany) polymethyl methacrylate (PMMA) embedding system optimized for hard tissues. Fixed samples were dehydrated through graded ethanol (70–100%), infiltrated in the cold-polymerizing resin according to manufacturer instructions, and embedded under vacuum at −4 °C. Blocks were sectioned using a precision diamond saw (Exakt system) to obtain 150–200 µm sections, which were then ground and polished to 40–60 µm using diamond abrasive sheets. Selected sections were mounted onto glass slides with Technovit^®^ 7200 (Kulzer, Hanau, Germany) [37]. Masson Trichrome stains were performed to assess bone formation and matrix organization. Some sections were deplasticized and demineralized before staining with Sirius Red, to objectivate mature collagen bundles. Slides were digitized using a ZEISS Axio Scan. Z1 slide scanner (Zeiss, Oberkochen, Germany).

### 2.6. Statistical Analysis

Quantitative data are reported as mean ± standard error of the mean. Post-acquisition image processing and quantitative analyses of µCT datasets were performed using ImageJ software (FiJi version, GNU General Public License v3). For each vertebra, quantitative measurements were obtained from 20 consecutive µCT slices selected from the inner region of the regenerated defect, in order to minimize edge effects and ensure consistent assessment of newly formed mineralized tissue. Statistical analyses were conducted using SigmaStat software (Version 3.0, Inpixon HQ, Palo Alto, CA, USA).

The experimental design involved six rats, each bearing four critical-size defects corresponding to the different experimental conditions, such that each animal served as its own internal control. For statistical purposes, the animal was considered the experimental unit, and group comparisons were therefore performed with *n* = 6 per condition. For each experimental group (control, nacre, pSi microparticles, and nacre–pSi composite), quantitative data derived from the six animals were pooled to minimize inter-animal variability.

Prior to analysis, data distribution was assessed for normality. When normality assumptions were met, comparisons between groups were performed using parametric one-way analysis of variance (ANOVA). When normality tests failed, non-parametric Kruskal–Wallis one-way analysis of variance on ranks was applied. Statistical significance was set at *p* < 0.05. Given the limited sample size and the proof-of-concept nature of the study, statistical analyses were considered exploratory and were intended to support observed trends rather than to provide definitive quantitative comparisons.

## 3. Results

### 3.1. Micro-CT Assessment of Bone Regeneration

**Figure 2:**
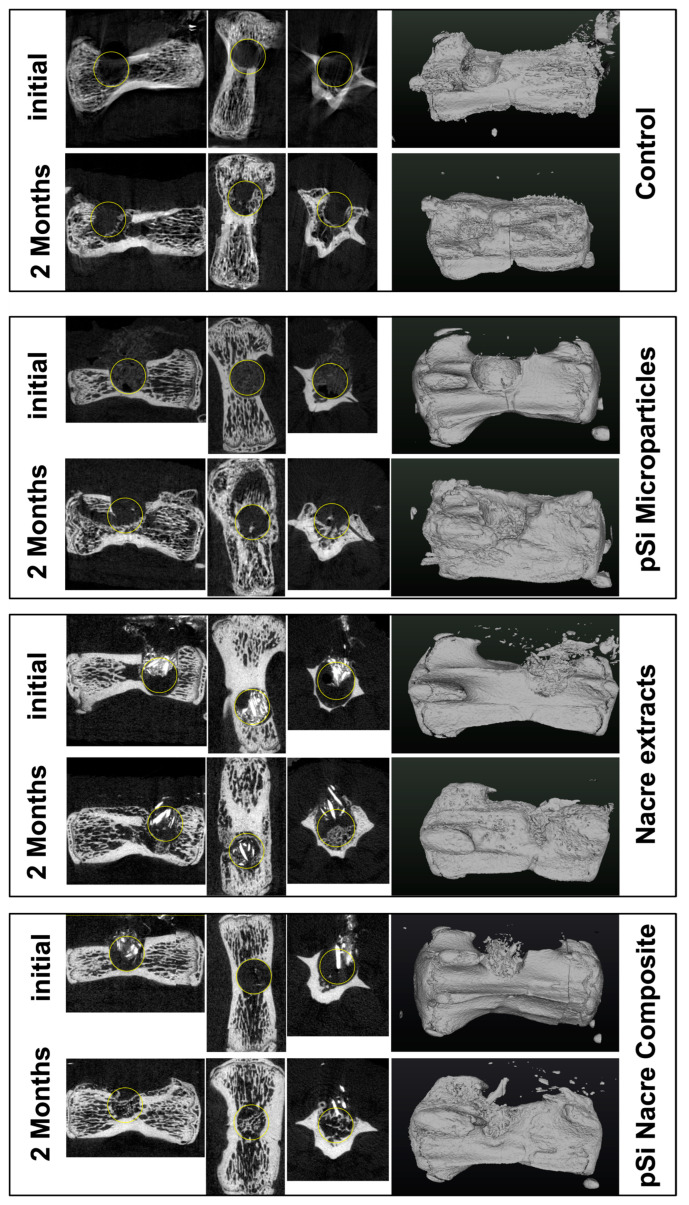
Micro Computerized Tomography of vertebrae, immediately after surgery, and 2 months after healing. Each line presents longitudinal slice, horizontal slice, axial slice, and 3D reconstruction. Yellow circle is added to highlight the drilled bone defect.

At 60 days post-implantation, micro-CT analysis revealed clear differences in bone repair among the four treatment conditions (Figure 2). Empty defects (control) showed minimal spontaneous bone regeneration, with the defect region remaining largely radiolucent and unbridged. In contrast, defects filled with nacre particles alone or oxidized porous silicon microparticles (pSi-MP) exhibited partial bone formation, characterized by scattered mineralized islands within the defect.

**Figure 3:**
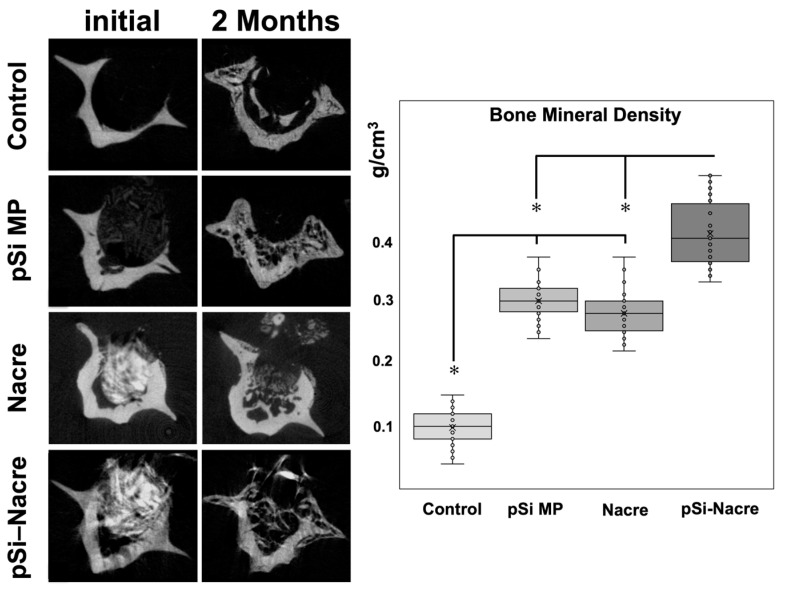
Micro Computerized Tomography and Bone Mineral Density (BMD) measurements. Histogram presents BMD values as measured on µCT slices after 2 months of healing. (*) represents significant difference (*p* < 0.05) between groups.

The most pronounced regeneration was observed in defects treated with the nacre + pSi-MP composite, which showed near-complete defect closure. Newly formed mineral bridged the defect and reached densities comparable to those of the surrounding native bone. Quantitative micro-CT analysis demonstrated that the bone mineral density (BMD) of composite-treated vertebrae was significantly higher than that of nacre-only or pSi-only groups, and markedly greater than the empty controls (Figure 3). The BMD values of the nacre-only and pSi-MP groups were comparable, indicating that each material independently supported partial osteogenesis.

### 3.2. Histological Evidence of Mineralized Tissue Formation

**Figure 4:**
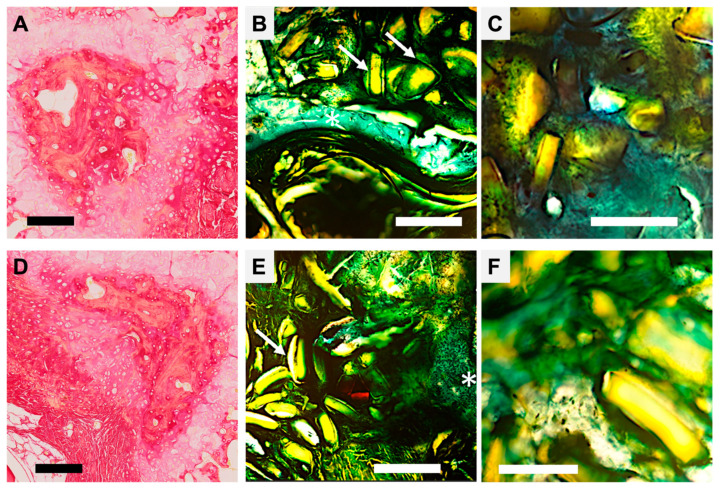
Histological section of vertebrae after healing, with Sirius Red and Goldner Masson staining. (**A**–**C**) Regeneration with pSi microparticles: (**A**) Decalcified sample, Red Sirius staining, Magnification ×5, Scale bar = 200 µm. Psi particle appears included in mineralizing collagen structures (in dark red). (**B**) Goldner Masson staining, Magnification ×5, Scale bar = 200 µm. White arrows point to dissolving pSi particles covered by green connective tissue, in proximity to the regenerating bone (white asterisk). (**C**) Goldner Masson staining, Magnification ×10, scale bar = 50 µm. Integration of the pSi particle closely attached to forming bone without inflammatory tissue. (**D**–**F**) Regeneration with nacre-based materials: (**D**) Decalcified sample, Red Sirius staining, Magnification ×5, Scale bar = 200 µm. The remaining nacre particle is surrounded by mineralizing collagen structures (in dark red). (**E**) Goldner Masson staining, Magnification ×5, Scale bar = 200 µm. White arrows points nacre particles covered by green connective tissue, white asterisk shows regenerating bone. (**F**) Goldner Masson staining, Magnification ×10, scale bar = 50 µm. Particles are closely attached to forming bone without inflammatory tissue.

Histological analysis of decalcified and non-decalcified sections (Technovit 9100 embedding) confirmed the radiographic findings (Figure 4). In the control defects, a fibro-cellular tissue filled the cavity, with no evidence of bridging woven or lamellar bone. In the nacre and pSi-MP groups, newly formed woven bone extended inward from the defect edges; however, central regions remained incompletely filled.

In defects treated with the nacre–pSi composite, a continuous rim of mineralized tissue was observed spanning the entire defect. The newly formed bone exhibited a dense matrix with active osteoblast lining and an organized trabecular structure. Backscattered electron microscopy further confirmed a highly mineralized bone phase, with incomplete mineralization only at the peripheral woven bone rim.

### 3.3. Material–Tissue Integration and Local Biocompatibility

Both nacre particles and pSi-MP were found closely apposed to newly formed bone without evidence of fibrous encapsulation or inflammatory infiltrate (Figure 4). Particles were embedded within the mineralized matrix, and osteogenic cells were observed in direct contact with material surfaces. This intimate interface suggests excellent osteointegration of each biomaterial.

In the composite group, nacre and pSi particles were consistently integrated within the regenerating bone, and the transition between the material and newly deposited matrix was seamless. No adverse reactions, necrosis, or inflammatory tissue were detected in any sample at 60 days.

### 3.4. Comparative Analysis of Bone Regeneration

A comparison of all groups is shown in Figure 3.

- Controls consistently displayed the lowest BMD and no defect bridging.
- Nacre and pSi-MP groups demonstrated similar intermediate levels of bone regeneration.
- The nacre + pSi composite group showed the highest BMD values and the most complete structural repair.

These findings indicate a synergistic effect when nacre-based materials and porous silicon microparticles are combined, resulting in enhanced mineral deposition and more consistent defect bridging compared with either material alone.

## 4. Discussion

The present study demonstrates that natural nacre-based materials significantly enhance bone regeneration in a rat vertebral critical-size defect model, particularly when combined with oxidized porous silicon microparticles (pSi-MP). While nacre or pSi alone supported partial repair, their composite induced near-complete defect bridging, higher mineral density, and seamless integration with host bone. These findings align with and extend earlier observations that nacre’s organic matrix contains potent osteogenic cues, and they introduce a previously unexplored synergy between nacre-based materials and silicon-based bioactive carriers.

### 4.1. Interpretation of Bone Mineral Density (BMD) in the Presence of Mineral Biomaterials

Interpretation of µCT-derived bone mineral density values in the present study requires particular caution, as the implanted materials themselves contribute to X-ray attenuation. As previously discussed in similar vertebral defect models using porous silicon-based biomaterials, µCT measurements in such contexts reflect an apparent mineral density rather than a direct and exclusive quantification of newly formed bone. Residual mineral phases from nacre (calcium carbonate) and oxidized porous silicon are therefore expected to contribute to the attenuation signal, particularly at early and intermediate stages of healing, when material resorption and tissue remodeling are still ongoing [32].

Importantly, however, the validity of the present findings does not rely solely on absolute BMD values. First, all experimental groups were analyzed under identical acquisition, reconstruction, and segmentation conditions, allowing relative comparisons between treatments within the same model. Second, the temporal and spatial patterns observed by µCT—including defect bridging, continuity of mineralized tissue, and progressive integration with host bone—were consistent with histological observations, which demonstrated new bone formation extending from defect margins and intimate contact between regenerated tissue and the implanted materials. Third, similar to previous observations with pSi-based scaffolds, increases in apparent mineral density over time coincided with progressive material resorption and replacement by newly formed bone, rather than with persistent inert mineral filling.

Taken together, these elements indicate that while µCT-derived BMD values in this study cannot be interpreted as a pure measure of bone mass, they remain a meaningful indicator of the overall mineralized tissue evolution within the defect when integrated with qualitative imaging and histological analyses. This combined interpretation supports the conclusion that the nacre–pSi composite promotes a more advanced regenerative process compared with individual components, despite the intrinsic radiopacity of the biomaterials involved.

### 4.2. Biological Rationale: Instructive Cues for Stem Cell-Mediated Osteogenesis

In the present study, bone regeneration was strictly dependent on the presence of bioactive and structural cues capable of supporting mesenchymal stem cell (MSC) recruitment and differentiation within the defect. Untreated control defects remained largely unrepaired after 60 days, indicating that endogenous signaling alone was insufficient to trigger robust osteogenesis in this vertebral critical-size model. In contrast, implantation of nacre particles or oxidized porous silicon microparticles resulted in partial bone formation, consistent with previous observations that MSCs can undergo osteogenic differentiation when provided with an appropriate bone-like microenvironment, even in the absence of exogenous growth factors [38,39,40]. Importantly, the combination of nacre and pSi produced a markedly enhanced regenerative response, suggesting that the convergence of biochemical and structural cues generated a local microenvironment more permissive to sustained osteogenesis. These findings support the concept that effective bone regeneration requires not a single instructive signal, but rather the integration of multiple complementary cues that collectively promote MSC adhesion, differentiation, and matrix mineralization.

### 4.3. Role of Nacre-Derived Components in Osteoinduction and Tissue Integration

Within this composite system, nacre-derived material played a central role in promoting bone formation and tissue integration [1,2,3]. In defects treated with nacre alone, bone formation extended into the defect area and newly formed tissue was observed in direct contact with nacre particles, indicating in situ bioactivity and osteoconductive behavior. This observation is consistent with previous reports describing the ability of nacre organic components to modulate osteogenic cell behavior, including enhanced osteoblast adhesion, proliferation, and expression of osteogenic markers [5,6,7,9,10,11,12,21]. In the present model, the mineral phase of the nacre-based material was considered an integral component of the biomimetic design, contributing to osteoconduction and radiodensity rather than representing a confounding factor. These effects translated into localized bone deposition along nacre particle surfaces, suggesting that nacre-derived components provided both a permissive substrate for cell attachment and biologically active cues supporting osteogenic differentiation. The mineral phase of the nacre-based material was considered an integral component of the biomimetic design, contributing to osteoconduction and radiodensity rather than representing a confounding factor.

Despite these favorable interactions, the regenerated tissue remained predominantly woven bone at 60 days, indicating that maturation toward lamellar bone had not yet occurred, as also reported in earlier in vivo studies using nacre-based materials [22,23]. This observation highlights the temporal limitations of the present study and suggests that longer healing periods or mechanically loaded models would be required to assess subsequent remodeling and long-term structural maturation of nacre-induced bone.

Our results support these previously described osteogenic activities: in defects treated with nacre, bone formation extended into the defect and integrated directly with particle surfaces. Nacre particles appeared “bioactive” in situ, showing no evident inflammatory reaction and supporting deposition of new bone in intimate contact with their surface. The present findings reinforce the concept that nacre proteins provide both passive and active regenerative contributions:

- Passive: their micro-particulate structure acts as a substrate for cell adhesion and Ca/P nucleation.
- Active: their soluble organic components stimulate osteogenic signaling pathways.

### 4.4. Bioactivity of Porous Silicon and Its Synergy with Nacre

Porous silicon is increasingly recognized as a bioactive, biodegradable, and osteoconductive biomaterial, attributable to its controlled release of silicic acid, a natural metabolite involved in collagen synthesis and bone mineralization. Silicon-based biomaterials accelerate osteogenesis, modulate osteoblast activity, and improve mineral deposition in vivo. Oxidized porous silicon, in particular, demonstrates slower biodegradation and sustained ion release, promoting prolonged bioactivity [31].

In our study, pSi-MP alone produced moderate bone regeneration and integrated into the forming tissue. These results align with previous observations that pSi microparticles support mineralized tissue formation while maintaining excellent biocompatibility.

The striking finding is that the nacre–pSi composite performed better than either component alone, suggesting a synergistic mechanism. Several hypotheses may explain this synergy:

- Complementary biochemical cues: Nacre proteins stimulate osteogenic differentiation, whereas silicic acid enhances collagen deposition and mineralization.
- Improved protein presentation: The porous structure of pSi may act as a carrier, trapping nacre proteins and releasing them gradually near osteoprogenitor cells.
- Enhanced nucleation: Both materials provide ion-rich microenvironments that favor Ca/P crystallization.
- Balanced degradation profiles: Oxidized pSi degrades slowly, maintaining structural support while nacre proteins rapidly initiate osteogenesis.

Together, these effects explain the nearly complete healing observed in the composite group and suggest that hybrid nacre–silicon materials may represent a promising new class of osteogenic biomaterials.

Beyond material chemistry, the present findings also highlight the critical role of material structure in governing bioactivity, a concept well established in the field of bioactive glass-based bone regeneration. Extensive in vivo studies on bioactive glass scaffolds have demonstrated that osteogenic performance is not solely dictated by composition, but is strongly influenced by microstructural features such as porosity, pore interconnectivity, surface reactivity, and degradation kinetics, which collectively regulate ion release, protein adsorption, and cell infiltration. Variations in scaffold architecture and dissolution behavior can profoundly affect bone ingrowth, vascularization, and the balance between osteoconduction and osteoinduction in vivo [41].

In this context, the nacre–pSi composite investigated in the present study shares conceptual parallels with bioactive glass microarchitectures, despite fundamental differences in material chemistry. Porous silicon microparticles provide a high-surface-area, degradable framework capable of controlled silicic acid release, while the nacre-derived organic/mineral fraction contributes biomimetic biochemical cues. The combination of these features likely creates a multifunctional microenvironment in which structural support, degradation-driven signaling, and biologically active components act in concert to promote bone formation. Similarly to observations reported for bioactive glass scaffolds, this synergy between structure and bioactivity appears to be a key determinant of the enhanced regenerative response observed in vivo, supporting the broader paradigm that rational structural design is central to achieving effective bone regeneration.

### 4.5. Osteogenic Behavior of Nacre

The osteogenic response observed in the present study is firmly rooted in a body of pioneering work conducted in the early 1990s by Lopez and colleagues, who first demonstrated that nacre is not an inert biomineral but an active inducer of bone formation. Using human osteoblast cultures, these studies showed that nacre chips were capable of attracting osteoblasts, promoting their organization into dense cellular bundles, and inducing the formation of mineralized, bone-like nodules in the absence of classical chemical osteogenic supplements [42,43]. Ultrastructural analyses further revealed a complete sequence of osteoblastic differentiation, including matrix vesicle release, collagen mineralization, and the emergence of woven bone-like structures, establishing nacre as a true osteoinductive substrate rather than a passive scaffold [43]. These in vitro observations were rapidly extended to in vivo and translational contexts. Early clinical and preclinical studies reported that crushed nacre powder, mixed with autologous blood and implanted into periodontal or maxillary bone defects, supported progressive bone regeneration without evidence of toxicity or inflammatory rejection, highlighting its remarkable biocompatibility and regenerative potential [44]. Subsequent large-animal investigations in sheep further demonstrated that solid nacre implants could integrate directly with host bone over long implantation periods, forming a stable and intimate bone–nacre interface characterized by active osteogenesis and Ca/P continuity at the interface [45]. Together, these seminal works established the concept that nacre acts as a biologically active, signal-bearing biomaterial capable of stimulating bone formation through both its organic matrix and mineral phase. This encouraging biocompatibility and osteogenic response was highlighted and contextualized by Westbroek and Marin in a seminal *Nature* commentary, which emphasized both the biological significance of nacre–bone interactions and their deep historical roots, notably recalling archaeological evidence of nacre dental implants integrated into Mayan jawbones [46].

The present study builds directly upon this foundational body of work by revisiting nacre-driven osteogenesis within a contemporary biomaterials framework. While the earlier studies primarily focused on nacre as a standalone material, our results demonstrate that its osteogenic activity can be further modulated and enhanced when combined with a degradable inorganic carrier such as oxidized porous silicon. In this sense, our findings do not challenge the conclusions of these pioneering investigations, but rather extend them by showing how nacre-based materials can be integrated into composite systems designed to control spatial presentation, degradation kinetics, and mineralized tissue evolution in a critical-size defect model. This continuity between early biological discoveries and modern biomimetic design underscores the enduring relevance of nacre as a template for bone regenerative strategies.

### 4.6. Biocompatibility and Host Response

With respect to local tissue response, standard non-decalcified histological sections did not reveal overt signs of acute or chronic inflammation at the implantation sites, such as extensive inflammatory cell infiltration, fibrous encapsulation, or necrotic tissue areas. The surrounding tissues appeared well integrated with the implanted materials, suggesting acceptable local biocompatibility under the experimental conditions used. However, it is important to emphasize that the absence of overt inflammatory features on routine histological staining does not allow definitive conclusions regarding immunological or inflammatory processes. The present study did not include dedicated immunohistochemical analyses or quantitative assessments of inflammatory markers, nor longitudinal evaluation of early inflammatory phases. As such, subtle or transient inflammatory responses cannot be excluded. This represents a limitation of the study and highlights the need for future investigations incorporating targeted immunological analyses to more comprehensively assess host–material interactions.

### 4.7. Relevance to the Broader Nacre Literature and Comparison with BMP-Based Strategies

Numerous recent advances in nacre-inspired biomaterials [15,16,24,25,26,27] emphasize the translation of nacre’s hierarchical structural logic into synthetic scaffolds. These studies consistently report enhanced osteogenesis, increased angiogenesis, immunomodulatory effects, improved mechanical properties, and favorable cell–material interactions.

Our work differs fundamentally from these biomimetic approaches by focusing on the natural nacre organic matrix, rather than its structural mimicry. The demonstration that nacre’s native macromolecules exert measurable osteogenic effects in vivo fills a major knowledge gap in the field and supports the idea that nacre is not only a structural inspiration but also a reservoir of osteoinductive biological molecules.

Bone morphogenetic proteins (BMP), particularly BMP-2 and BMP-7, are among the most potent osteoinductive factors known and have demonstrated robust bone-forming capacity in both preclinical and clinical settings. However, their clinical use remains restricted to limited indications due to the need for supraphysiological doses, rapid diffusion from implantation sites, high cost, and well-documented adverse effects, including ectopic bone formation, inflammation, and dose-dependent complications [47]. Recent work has emphasized that effective BMP-based bone regeneration requires not only precise dosing but also tightly controlled spatial and temporal delivery, often necessitating sophisticated carrier systems to mitigate these limitations. In contrast, nacre-derived materials do not aim to replicate the potency or specificity of recombinant BMPs. Instead, nacre proteins represent a naturally evolved, multi-component bioactive system that provides moderate but sustained osteogenic stimulation through synergistic biochemical and structural cues. Unlike BMPs, nacre proteins are embedded within a mineral-organic matrix that enables gradual release and local presentation of osteogenic signals, potentially reducing the risk of excessive or uncontrolled tissue responses. Their activity appears to rely on the combined modulation of osteoblast differentiation, matrix mineralization, and osteoclast regulation, rather than on strong single-pathway induction.

From a translational perspective, these differences suggest complementary rather than competing strategies. BMP-based therapies are highly effective when precisely delivered but are associated with regulatory, economic, and safety constraints. Nacre-derived materials, by contrast, offer a biomimetic and potentially safer alternative for enhancing bone regeneration through physiologically inspired signaling, without the need for exogenous growth factors. While their osteogenic efficacy is lower than that of BMPs in absolute terms, nacre proteins may be particularly attractive in applications where controlled, gradual bone formation and long-term biocompatibility are prioritized. The present findings support this concept by demonstrating that nacre-derived cues, especially when combined with a degradable inorganic carrier such as porous silicon, can promote substantial bone regeneration without relying on high-dose recombinant growth factors.

### 4.8. Limitations and Future Directions

Although the present findings are encouraging, several limitations of this study should be acknowledged. First, the regenerated tissue observed at 60 days consisted predominantly of woven bone, indicating that longer-term studies are required to assess subsequent remodeling toward lamellar or cortical bone. In addition, the critical-size rat vertebral defect model used here is non-load bearing; therefore, future investigations in load-bearing models (such as femur, tibia, or mandible) will be necessary to evaluate functional integration and mechanical competence.

Importantly, the present study did not include a positive control group, such as an autograft or a clinically approved bone substitute. This choice reflects the exploratory, proof-of-concept nature of the work, which was designed to compare the relative regenerative effects of nacre-derived material, porous silicon microparticles, and their combination within the same experimental framework. Nevertheless, the absence of a positive control limits direct comparison with current clinical standards and should be considered when interpreting the translational relevance of the findings.

Also, the study was not designed as a dose-optimization investigation, and this point could be an important perspective for future work. Furthermore, the exact molecular identity of the osteogenic nacre-derived components remains incompletely defined, and proteomics-based characterization will be required to better elucidate the specific macromolecules involved. The use of nacre-derived material in its native organic/mineral context therefore represents a deliberate methodological choice rather than an experimental oversight, and is acknowledged as a limitation of the present study.

Finally, although no overt inflammatory reaction was observed in standard histological sections, dedicated immunological analyses were not performed and subtle or transient inflammatory responses cannot be excluded. Future studies incorporating longer follow-up periods, mechanistic analyses, and evaluation in large-animal models will be essential before considering translation toward dental or orthopedic applications. In addition, age-related or osteoporotic conditions are expected to significantly influence the regenerative response by altering stem cell availability, osteogenic potential, vascularization, and remodeling kinetics. Assessing the performance of nacre–pSi composites in aged or osteoporotic animal models will therefore be an important step to determine the robustness and clinical relevance of this biomimetic strategy under compromised bone-healing conditions. Despite these limitations, the consistent material–tissue integration and enhanced mineralized tissue formation observed with the nacre–pSi composite support its potential as a biomimetic platform for further development in bone regenerative strategies.

## 5. Conclusions

This study demonstrates that natural nacre-derived materials exhibit intrinsic osteogenic activity capable of supporting bone regeneration in a vertebral critical-size defect model. While nacre particles and oxidized porous silicon microparticles each supported partial healing, their combination yielded a markedly enhanced regenerative response, characterized by near-complete defect bridging, increased apparent mineral density, and good material–tissue integration. These findings suggest a synergistic effect in which porous silicon provides a bioactive, resorbable scaffold with controlled silicic acid release, while nacre-derived components contribute biomimetic osteogenic cues that support early bone formation. Although the newly formed tissue remained predominantly woven bone at 60 days, no overt inflammatory reaction was observed in standard histological sections, and robust mineralized tissue deposition was evident, suggesting a favorable healing trajectory under the experimental conditions used. Together, these results identify the nacre–pSi composite as a promising proof-of-concept biomaterial for bone repair, warranting further investigation in long-term, mechanistic, and large-animal studies.

## Data Availability

Dataset available on request from the authors.

## Abbreviations

The following abbreviations are used in this manuscript:

BMD	Bone Mineral Density
MP	Microparticle
pSi	Porous silicon
MSC	Mesenchymal Stem Cell
